# Antibiotic Class and Outcome in Post-stroke Infections: An Individual Participant Data Pooled Analysis of VISTA-Acute

**DOI:** 10.3389/fneur.2019.00504

**Published:** 2019-05-14

**Authors:** Craig J. Smith, Calvin Heal, Andy Vail, Adam R. Jeans, Willeke F. Westendorp, Paul J. Nederkoorn, Diederik van de Beek, Lalit Kalra, Joan Montaner, Mark Woodhead, Andreas Meisel

**Affiliations:** ^1^Greater Manchester Comprehensive Stroke Centre, Manchester Academic Health Science Centre, Salford Royal NHS Foundation Trust, Salford, United Kingdom; ^2^Division of Cardiovascular Sciences, School of Medical Sciences, University of Manchester, Manchester, United Kingdom; ^3^Centre for Biostatistics, Manchester Academic Health Science Centre, University of Manchester, Manchester, United Kingdom; ^4^Division of Clinical Support Services and Tertiary Medicine, Department of Microbiology, Salford Royal NHS Foundation Trust, Salford, United Kingdom; ^5^Department of Neurology, Amsterdam Neuroscience, Academic Medical Center, University of Amsterdam, Amsterdam, Netherlands; ^6^Clinical Neurosciences, King's College Hospital NHS Foundation Trust London, London, United Kingdom; ^7^Neurovascular Research Laboratory, Vall d' Hebron Institute of Research, Barcelona, Spain; ^8^Stroke Research Program, Department of Neurology, Institute de Biomedicine of Seville, Hospital Universitario Virgen Macarena, IBiS/Hospital Universitario Virgen del Rocío/CSIC/University of Seville, Seville, Spain; ^9^Faculty of Biology, Medicine and Health, Manchester Academic Health Science Centre, University of Manchester, Manchester, United Kingdom; ^10^Department of Neurology, NeuroCure Clinical Research Center, Center for Stroke Research Berlin, Charité Universitaetsmedizin Berlin, Berlin, Germany

**Keywords:** stroke, acute, post-stroke infections, post-stroke pneumonia, antibiotics, prognosis

## Abstract

**Introduction:** Antibiotics used to treat post-stroke infections have differing antimicrobial and anti-inflammatory effects. Our aim was to investigate whether antibiotic class was associated with outcome after post-stroke infection.

**Methods:** We analyzed pooled individual participant data from the Virtual International Stroke Trials Archive (VISTA)-Acute. Patients with ischemic stroke and with an infection treated with systemic antibiotic therapy during the first 2 weeks after stroke onset were eligible. Antibiotics were grouped into eight classes, according to antimicrobial mechanism and prevalence. The primary analysis investigated whether antibiotic class for any infection, or for pneumonia, was independently associated with a shift in 90 day modified Rankin Scale (mRS) using ordinal logistic regression.

**Results:** 2,708 patients were eligible (median age [IQR] = 74 [65 to 80] y; 51% female; median [IQR] NIHSS score = 15 [11 to 19]). Pneumonia occurred in 35%. Treatment with macrolides (5% of any infections; 9% of pneumonias) was independently associated with more favorable mRS distribution for any infection [OR (95% CI) = 0.59 (0.42 to 0.83), *p* = 0.004] and for pneumonia [OR (95% CI) = 0.46 (0.29 to 0.73), *p* = 0.001]. Unfavorable mRS distribution was independently associated with treatment of any infection either with carbapenems, cephalosporins or monobactams [OR (95% CI) = 1.62 (1.33 to 1.97), *p* < 0.001], penicillin plus β-lactamase inhibitors [OR (95% CI) = 1.26 (1.03 to 1.54), *p* = 0.025] or with aminoglycosides [OR (95% CI) = 1.73 (1.22 to 2.46), *p* = 0.002].

**Conclusion:** This retrospective study has several limitations including effect modification and confounding by indication. Macrolides may have favorable immune-modulatory effects in stroke-associated infections. Prospective evaluation of the impact of antibiotic class on treatment of post-stroke infections is warranted.

## Introduction

Infections frequently complicate stroke, occurring in up to 30% of patients, and increase the likelihood of death and unfavorable outcomes in survivors (1–3). Whilst antibiotics are the mainstay of treatment, the microbiological etiology of common infections complicating stroke, such as pneumonia or urinary tract infection, are poorly characterized. Further, there are no antibiotic treatment trials of infections complicating stroke. The effectiveness of different antibiotic classes is therefore uncertain, there is a lack of evidence to inform antibiotic guidelines (e.g., for pneumonia complicating stroke) and empirical antibiotic treatment is variable (4, 5).

Inflammatory and immune responses play a central role in the pathophysiology of stroke and associated clinical outcomes (6). Post-stroke infections exacerbate deleterious inflammatory and immune responses, which may impact further on adverse outcomes (7). Antibiotics used to treat post-stroke infections can modulate the pathophysiology of experimental stroke independent of their anti-microbial effects, by modulating inflammatory or excitotoxic pathways (8–14). Randomized trials of prophylactic antibiotics in acute stroke have failed to improve clinical outcomes or prevent pneumonia (3, 5, 15–17), and had varying effects in preventing urinary tract infections. This has raised questions about the potential effectiveness of some antibiotic classes commonly used for post-stroke infections, particularly pneumonia (18).

Taken together, these data suggest that choice of antibiotic class for post-stroke infections could have important implications for clinical outcomes. We therefore hypothesized that antibiotic class influences outcome after stroke relating to spectrum of antimicrobial coverage and to other (e.g., inflammatory) mechanisms independent of antimicrobial effects. The aim of this study was to investigate whether class of antibiotic used to treat clinically diagnosed pneumonia or any infection in the first 2 weeks after stroke was associated with clinical outcomes.

## Patients and Methods

We undertook a retrospective pooled analysis of individual participant data from the Virtual International Stroke Trials Archive (VISTA)-Acute (www.virtualtrialsarchives.org/vista). The study protocol was approved by the VISTA-Acute Steering Committee. Dedicated ethical approval was not required as anonymized historical patient data were being used. The VISTA-Acute repository was screened by the VISTA coordinator for eligible acute ischemic stroke randomized controlled trials (RCTs) meeting the following study-level criteria: (i) Adverse Event (AE) and Serious Adverse Event (SAE) data recorded during the first 2 weeks after enrolment which included post-stroke infections, including both type of infection and name of antibiotic therapy; (ii) 90 day clinical outcomes: modified Rankin Scale (mRS), National Institutes of Health Stroke Scale (NIHSS) and Barthel Index (BI); (iii) core demographic variables (including age, sex, country where randomized, year of study, vascular risk profile and smoking history) and clinical variables (baseline NIHSS, baseline mRS pre-stroke, concurrent medications, baseline glucose concentration). For the eligible RCTs identified, individual participant data for trial participants with an infection recorded and treated within the first 2 weeks after stroke onset were anonymized and extracted. Individual participant SAE and AE data were reviewed by a single researcher (CJS) to classify infection sub-type and categorize systemic antibiotic treatment by class, blinded to the baseline characteristics and clinical outcomes.

### Infection Category

Categorization of the infections into sub-types was based on review of the information recorded in the reported SAEs and AEs, and was therefore necessarily both pragmatic and also reflective of usual clinical practice. Infections were categorized as follows: (i) Pneumonia (including description or coding of pneumonia, aspiration pneumonia, inhalation pneumonia, chest infection, lung consolidation, bronchopulmonary infection, bronchial infection); (ii) Tracheobronchitis (including acute bronchitis, acute exacerbation of bronchitis, acute tracheobronchitis, upper respiratory tract infection, exacerbation of chronic airways disease/obstructive pulmonary disease or asthma); (iii) Urogenital infections (including urinary tract infection, pyelonephritis, cystitis, prostatitis, vaginal infection); (iv) Other defined infections (skin and soft tissue, dental, joint, gastro-intestinal, meningitis/ ventriculitis, pharyngeal/throat, and ear) requiring systemic antibiotics. The majority of these were self-explanatory based on reported diagnosis (e.g., aspiration pneumonia, acute bronchopneumonia, hospital-acquired pneumonia; acute tracheobronchitis, exacerbation of chronic bronchitis; acute pyelonephritis, urinary tract infection) or description (e.g., “yellow secretions, basilar crackle + scattered rhonchi”; “infection intravenous cannula site”). A final category, (v) Uncertain source of infection, was included for when there was sufficient clinical suspicion of infection to initiate systemic antibiotics but without a definite source of infection reported or where the site of infection could not be concluded from the available information. Patients were excluded if they received preventative antibiotics; had an identified infection but received no systemic antibiotic treatment, received treatment for an infection caused by proven or suspected *Candida* spp or *Mycobacterial* spp. Uncertainties relating to the site of infection or any other aspect of categorization into sub-type were resolved by discussion with the study microbiologist (ARJ).

### Antibiotic Class

All recorded antibiotics were individually reviewed by a single researcher (CJS). Antibiotics with systemic (oral, via enteral tube, intravenous or intramuscular) administration were grouped, prior to consideration of their association with outcomes, as follows based on mechanism of action (Table 1) and prevalence in the final cohort: (i) lincosamides and tetracyclines; (ii) cephalosporins, carbapenems, and monobactams; (iii) aminoglycosides; (iv) fluoroquinolones; (v) penicillins; (vi) penicillin plus β-lactamase inhibitor; (vii) other (sulphonamides, folic acid inhibitors, metronidazole, nitrofurans, and glycopeptides); (viii) macrolides.

**Table 1:** Antibiotics and spectrum of activity.

	***Staphylococcus aureus*, MSSA**	***Staphylococcus aureus*, MRSA**	***Streptococcus pneumoniae***	**Enterococci**	**Coliforms**	**Coliforms, ESBL**	***Haemophilus influenzae***	***Pseudomonas***	***Acinetobacter***	**Anaerobes (Gram positive)**	**Anaerobes (Gram negative)**	***Mycoplasma***	**Site of action**
Aminoglycosides	+	+	0	0	+	+	0	+	+	0	0	0	Ribosome
Carbapenems	++	0	++	0	++	++	++	+	+	+	++	0	Cell wall
Cephalosporins (3^rd^ generation)	+	0	++	0	±	0	++	+	±	+	0	0	Cell wall
Fluoroquinolones	+	±	±	±	+	+	++	+	+	0	0	+	Nucleic acid
Glycopeptides	+	+	+	+	0	0	0	0	0	±	0	0	Cell wall
Lincosamides	+	±	±	0	0	0	0	0	0	+	±	0	Ribosome
Macrolides	+	±	±	0	0	0	±	0	0	±	0	+	Ribosome
Metronidazole	0	0	0	0	0	0	0	0	0	±	++	0	Nucleic acid
Monobactams	0	0	0	0	+	0	+	+	0	0	0	0	Cell wall
Nitrofurans	0	0	0	+	+	+	0	0	0	0	0	0	Nucleic acid
Penicillins	++	0	+	±	±	–	±	0	0	++	0	0	Cell wall
Penicillin plus β-lactamase inhibitors	+	0	+	±	+	±	+	+	±	++	++	0	Cell wall
Sulphonamides/folic acid inhibitors	+	+	±	±	+	+	+	0	±	0	0	0	Folate synthesis
Tetracyclines	+	+	±	0	±	±	+	0	±	+	±	+	Ribosome

Where activity varies within an antibiotic group, the most active commercially available agent is assumed.

*0, Limited useful activity as monotherapy; ±, Variable activity. Efficacy often limited by resistance; +, Active. Likely to be clinically effective, but resistance may occur; ++, Highly active. Agent highly potent and resistance unusual*.

### Statistical Analyses

The exposure of interest was antibiotic class used to treat post-stroke infection during the first 2 weeks after ischemic stroke onset. The primary outcome measure was the 7-point mRS score at 90 days (incorporating death as score 6). Secondary outcomes included NIHSS and BI at 90 days. Baseline characteristics were summarized for pooled study data. To determine whether antibiotic class, or other prognostic variables were associated with the primary outcome, we performed multifactorial ordinal logistic regression to obtain an adjusted odds ratio with 95% confidence interval (CI) for a shift in mRS. Antibiotic class and infection subtype were each entered into the model as categorical predictor variables, allowing us to account for subsequent infections and multiple different antibiotic exposures in the analysis. Other potential confounding individual-level and study-level characteristics were also included as independent variables and included age, sex, year, geographical region/continent, baseline NIHSS, categorical infection subtype, thrombolysis treatment, and prior statin treatment. We included statins as they are independently associated with outcome after stroke (19) and are implicated in both the risk of stroke-associated infections and prognosis (20–23). Data restrictions prevented direct inclusion of a trial identifier in the analyses. In the primary analysis we investigated the independent association between antibiotic class and 90 day mRS both for any infection and also for pneumonia separately. Secondary analyses of 90 day BI and NIHSS incorporated deceased patients, and used ordinal logistic regression undertaken for any infection and also pneumonia separately, adjusted for the same independent variables.

### Sample Size

A preliminary screen of individual participant data for >30 RCTS in VISTA-Acute led us to anticipate inclusion of >2,000 participants meeting the eligibility criteria. We anticipated that statistical power would depend critically on the distribution of antibiotic classes used, and that of the mRS within patients treated for infection. Analysis on the ordinal scale, assuming the proportional odds assumption holds in eligible patients, as it would be anticipated to do in unselected patients, would increase power over a binary outcome analysis. For illustrative purposes, a simple power calculation on the binary scale indicated that a comparison of 600 patients treated with each of two classes of antibiotics would provide 80% power to detect an odds ratio of 1.5 from a baseline of 75% with “good outcome.”

## Results

2,708 patients were eligible with a total of 3,081 infections treated with systemic antibiotics during the first 2 weeks after stroke onset (any infection). Pneumonia occurred in 35%, and urogenital infections in 42% of the participants. Trials between 1998 and 2013 were represented, which included participating centers from Europe, Asia, Africa, Australasia and the Americas. The baseline characteristics and clinical outcomes for the final sample (median age [IQR] = 74 [65 to 80] y; 51% female; median [IQR] NIHSS score = 15 [11 to 19]; 90 day case fatality 26%) and the different infection categories are presented in Table 2. The baseline characteristics and clinical outcomes for patients receiving the different antibiotic classes in any infection and pneumonia are presented in Tables 3, 4.

**Table 2:** Baseline characteristics and 90 day clinical outcomes according to infection category.

**Number of infections**	**Any infection 3081**	**Pneumonia 958**	**Tracheobronchitis 170**	**Urogenital infection 1132**	**Other infection 330**	**Uncertain infection 491**
Number of patients	2708	958	170	1132	330	490
Median (IQR) age (y)	74 (65 to 80)	74 (66 to 80)	72 (65 to 78)	74 (66 to 80)	73 (64 to 80)	73 (65 to 79)
Female sex, *n* (%)	1372 (51%)	387 (40%)	67 (39%)	727 (64%)	140 (42%)	225 (46%)
Median (IQR) NIHSS	15 (11 to 19)	16 (12 to 20)	13 (9 to 17)	14 (11 to 18)	14 (10 to 17)	16 (12 to 20)
Median (IQR) time to first infection (d)	5 (3 to 9)	4 (2 to 7)	3 (2 to 7)	6 (3 to 14)	6 (3 to 21)	4 (2 to 6)
IV thrombolysis, *n* (%)	795 (26%)	270 (28%)	44 (26%)	282 (25%)	75 (23%)	124 (25%)
Mean (SD) pre-stroke mRS	1.2 (0.6)	1.2 (0.6)	1.1 (0.3)	1.3 (0.7)	1.3 (0.8)	1.2 (0.5)
Median (IQR) glucose concentration (mmol/L)	6.9 (5.8 to 8.6)	7.0 (5.9 to 9.0)	6.9 (5.7 to 9.1)	6.7 (5.7 to 8.2)	6.8 (5.8 to 8.4)	7.2 (6.0 to 8.7)
Prior statin, *n* (%)	210 (6%)	43 (4%)	10 (6%)	97 (9%)	32 (10%)	28 (6%)
**Vascular risk factors**, ***N*** **(%)**						
Previous stroke	558 (19%)	184 (20%)	35 (21%)	197 (18%)	64 (20%)	78 (17%)
Previous TIA	258 (9%)	83 (9%)	10 (6%)	96 (9%)	28 (9%)	41 (9%)
Diabetes mellitus	685 (22%)	231 (24%)	42 (25%)	218 (19%)	71 (22%)	123 (25%)
Hypertension	1994 (66%)	609 (64%)	115 (69%)	731 (66%)	213 (65%)	326 (67%)
Previous myocardial infarction	390 (13%)	142 (15%)	16 (10%)	136 (12%)	46 (14%)	50 (10%)
Atrial fibrillation	841 (29%)	302 (33%)	43 (27%)	277 (27%)	78 (26%)	141 (31%)
**Smoking status**						
Non-smoker	322 (10%)	94 (10%)	10 (6%)	133 (12%)	37 (11%)	48 (10%)
Smoker	495 (16%)	147 (15%)	19 (11%)	191 (17%)	63 (19%)	75 (15%)
**90 day outcomes, median (IQR)**						
mRS	4 (3 to 6)	5 (4 to 6)	4 (2 to 5)	4 (2 to 5)	4 (2 to 6)	4 (3 to 6)
BI	25 (0 to 80)	5 (0 to 55)	55 (0 to 95)	50 (5 to 85)	30 (0 to 85)	10 (0 to 70)
NIHSS	11 (4 to 42)	17 (7 to 42)	7 (3 to 42)	8 (3 to 16)	11 (3 to 42)	13 (5 to 42)
90 day case fatality, *n* (%)	794 (26%)	353 (37%)	39 (23%)	157 (14%)	84 (26%)	161 (33%)

*NIHSS, National Institutes of Health Stroke Scale; mRS, modified Rankin Scale; TIA, transient ischemic attack; BI, Barthel Index*.

**Table 3:** Baseline characteristics and 90 day outcomes according to antibiotic class exposure in any infection.

	**Lincosamides and tetracyclines**	**Cephalosporins, carbapenems and monobactams**	**Aminoglycosides**	**Fluoroquinolones**	**Penicillins**	**Penicillin plus β-lactamase inhibitors**	Other^*^	**Macrolides**
Number of patients	180	815	165	930	315	873	670	142
Median (IQR) age (y)	74 (63 to 80)	73 (65 to 80)	73 (67 to 79)	74 (66 to 80)	75 (65 to 80)	74 (65 to 80)	75 (67 to 81)	71 (62 to 79)
Female sex, *n* (%)	73 (41%)	363 (45%)	59 (36%)	505 (54%)	158 (50%)	385 (44%)	384 (57%)	61 (43%)
Median (IQR) NIHSS	17 (12 to 20)	15 (11 to 19)	16 (12 to 19)	14 (11 to 19)	15 (11 to 19)	15 (11 to 19)	15 (11 to 19)	13 (9 to 18)
Median (IQR) time to first infection (d)	4 (2 to 8)	5 (2 to 8)	6 (3 to 12)	5 (3 to 10)	7 (4 to 17)	4 (2 to 7)	6 (3 to 15)	5 (2 to 11)
IV thrombolysis, *n* (%)	43 (24%)	187 (23%)	30 (18%)	267 (29%)	73 (23%)	248 (28%)	179 (27%)	36 (25%)
Mean (SD) pre-stroke mRS	1.2 (0.7)	1.2 (0.6)	1.4 (0.8)	1.1 (0.5)	1.5 (0.9)	1.1 (0.6)	1.4 (0.8)	1.3 (0.7)
Median (IQR) glucose (mmol/L)	7.4 (5.9 to 9.1)	6.8 (5.8 to 8.9)	7.3 (6.1 to 8.5)	6.8 (5.9 to 8.4)	6.7 (5.8 to 8.6)	7.2 (5.9 to 9.2)	6.7 (5.8 to 8.3)	6.5 (5.7 to 7.9)
Prior statin, *n* (%)	8 (4%)	62 (8%)	11 (7%)	66 (7%)	22 (7%)	58 (7%)	60 (9%)	4 (3%)
**Vascular risk factors**, ***n*** **(%)**								
Previous stroke	33 (19%)	146 (19%)	31 (19%)	156 (17%)	50 (17%)	149 (18%)	148 (23%)	28 (20%)
Previous TIA	15 (9%)	62 (8%)	13 (8%)	77 (9%)	22 (7%)	68 (8%)	59 (9%)	9 (6%)
Diabetes mellitus	49 (27%)	189 (23%)	42 (25%)	195 (21%)	68 (22%)	222 (25%)	155 (23%)	23 (16%)
Hypertension	108 (60%)	518 (65%)	117 (71%)	607 (66%)	195 (64%)	557 (65%)	460 (69%)	69 (49%)
Previous myocardial infarction	25 (14%)	98 (12%)	31 (19%)	113 (12%)	35 (11%)	104 (12%)	87 (13%)	17 (12%)
Atrial fibrillation	60 (35%)	216 (29%)	47 (30%)	243 (28%)	92 (32%)	234 (28%)	172 (28%)	34 (25%)
**Smoking status**								
Non-smoker	23 (13%)	107 (13%)	14 (8%)	121 (13%)	39 (12%)	51 (6%)	56 (8%)	10 (7%)
Smoker	31 (17%)	153 (19%)	28 (17%)	160 (17%)	61 (19%)	105 (12%)	140 (21%)	27 (19%)
**90 day outcomes, median (IQR)**								
mRS	5 (4 to 6)	4 (3 to 6)	5 (4 to 6)	4 (3 to 5)	4 (3 to 5)	4 (3 to 6)	4 (3 to 6)	3 (2 to 5)
BI	0 (0 to 45)	10 (0 to 65)	0 (0 to 45)	40 (0 to 85)	25 (0 to 75)	20 (0 to 75)	15 (0 to 70)	65 (0 to 95)
NIHSS	19 (8 to 42)	14 (6 to 42)	20 (8 to 42)	9 (3 to 36)	11 (4 to 42)	12 (5 to 42)	12 (4 to 42)	7.5 (3 to 42)
90 day case fatality, *n* (%)	69 (39%)	271 (33%)	64 (39%)	203 (22%)	63 (20%)	264 (31%)	180 (27%)	32 (23%)

* Other includes sulphonamides/folic acid inhibitors, metronidazole, glycopeptides, and nitrofurans.

*NIHSS, National Institutes of Health Stroke Scale; mRS, modified Rankin Scale; TIA, transient ischemic attack; BI, Barthel Index*.

**Table 4:** Baseline characteristics and 90 day outcomes according to antibiotic class exposure in pneumonia.

	**Lincosamides and tetracyclines**	**Cephalosporins, carbapenems, and monobactams**	**Aminoglycosides**	**Fluoroquinolones**	**Penicillins**	**Penicillin plus β-lactamase inhibitors**	Other^*^	**Macrolides**
Number of patients	120	403	78	254	115	395	224	83
Median (IQR) age (y)	75 (64 to 80)	73 (64 to 80)	72.5 (64 to 80)	75 (67 to 80)	76 (66 to 81)	75 (67 to 81)	75 (67.5 to 81)	70 (64 to 79)
Female sex, *n* (%)	48 (40%)	159 (39%)	24 (31%)	97 (38%)	44 (38%)	161 (41%)	93 (42%)	37 (45%)
Median (IQR) NIHSS	17 (12 to 20)	16 (12 to 19)	16.5 (13 to 20)	16 (13 to 20)	16 (13 to 18)	16 (12 to 20)	16 (11 to 19)	14 (10 to 18)
Median (IQR) time to first infection (d)	4 (2 to 7)	4 (2 to 7)	5 (3 to 8)	4 (2 to 7.5)	6 (3 to 15)	4 (2 to 7)	5 (3 to 9)	3.5 (1 to 7)
IV thrombolysis, *n* (%)	27 (23%)	96 (24%)	13 (17%)	87 (34%)	29 (25%)	131 (33%)	65 (29%)	19 (23%)
Mean (SD) pre-stroke mRS	1.3 (0.7)	1.2 (0.5)	1.4 (0.8)	1.1 (0.5)	1.4 (0.8)	1.2 (0.6)	1.3 (0.8)	1.2 (0.7)
Median (IQR) glucose (mmol/L)	7.4 (6 to 9.7)	6.8 (5.7 to 8.9)	7 (5.8 to 8.4)	6.8 (5.9 to 8.9)	6.7 (5.9 to 8.6)	7.3 (5.9 to 9.8)	6.7 (5.7 to 8.3)	6.4 (5.7 to 8.2)
Prior statin, *n* (%)	3 (3%)	26 (6%)	3 (4%)	12 (5%)	7 (6%)	18 (5%)	13 (6%)	1 (1%)
**Vascular risk factors**, ***n*** **(%)**								
Previous stroke	22 (19%)	78 (21%)	13 (18%)	43 (17%)	25 (22%)	67 (17%)	46 (21%)	18 (22%)
Previous TIA	9 (8%)	30 (8%)	9 (12%)	25 (10%)	11 (10%)	34 (9%)	12 (5%)	5 (6%)
Diabetes mellitus	28 (23%)	96 (24%)	18 (23%)	64 (25%)	23 (20%)	106 (27%)	54 (24%)	11 (13%)
Hypertension	64 (54%)	248 (63%)	53 (68%)	169 (67%)	76 (67%)	252 (65%)	152 (68%)	37 (45%)
Previous myocardial infarction	19 (15%)	57 (14%)	12 (15%)	36 (14%)	19 (17%)	56 (14%)	31 (13%)	10 (12%)
Atrial fibrillation	44 (39%)	119 (32%)	22 (29%)	72 (30%)	44 (40%)	122 (33%)	69 (34%)	20 (24%)
**Smoking status**								
Non-smoker	14 (12%)	57 (14%)	7 (9%)	33 (13%)	9 (8%)	31 (8%)	15 (7%)	6 (7%)
Smoker	21 (18%)	79 (20%)	13 (17%)	54 (21%)	23 (20%)	52 (13%)	48 (21%)	14 (17%)
**90 Day outcomes, median (IQR)**								
mRS	5 (4 to 6)	5 (4 to 6)	5 (4 to 6)	5 (4 to 6)	4 (4 to 6)	5 (4 to 6)	5 (4 to 6)	4 (2 to 6)
BI	0 (0 to 35)	5 (0 to 50)	0 (0 to 25)	5 (0 to 45)	5 (0 to 40)	5 (0 to 55)	0 (0 to 35)	60 (0 to 95)
NIHSS	19 (8 to 42)	18 (7.5 to 42)	42 (10 to 42)	16 (7 to 42)	16 (8 to 42)	16 (7 to 42)	19 (8 to 42)	8 (3 to 42)
90 day case fatality, *n* (%)	45 (38%)	151 (38%)	32 (41%)	90 (36%)	32 (28%)	144 (37%)	85 (38%)	22 (27%)

* Other includes sulphonamides/folic acid inhibitors, metronidazole, glycopeptides, and nitrofurans.

*NIHSS, National Institutes of Health Stroke Scale; mRS, modified Rankin Scale; TIA, transient ischemic attack; BI, Barthel Index*.

The prevalence of the antibiotic classes in the different infection categories is shown in Table 5. Antibiotic dosage, duration and route of administration were not available. Table 6 shows the frequency of prescription of monotherapy with one antibiotic agent, or combination treatment with >1 antibiotic agent, for the infection categories. Combination therapy with >1 antibiotic agents occurred most often in pneumonia, with at least two antibiotics prescribed in 42% of cases compared with 20% in tracheobronchitis, 21% in urogenital infections and 33% in other infections.

**Table 5:** Prevalence of antibiotic classes for infection categories expressed as % of total number of antibiotic prescriptions.

**Number of patients**	**Any infection 2708**	**Pneumonia 958**	**Tracheobronchitis 170**	**Urogenital infection 1132**	**Other infection 330**	**Uncertain infection 490**
Number of antibiotic prescriptions	5237	1862	233	1723	650	769
Antibiotic class, *n* (%)						
**Lincosamides and tetracyclines**						
Tetracyclines	33 (0.6%)	10 (0.5%)	9 (3.9%)	1 (<0.1%)	13 (2%)	0
Lincosamides	186 (3.6%)	124 (6.7%)	3 (1.3%)	10 (0.6%)	15 (2.3%)	34 (4.4%)
**Cephalosporins, carbapenems, monobactams**						
Cephalosporins	986 (18.8%)	436 (23.4%)	45 (19.3%)	216 (12.5%)	125 (19.2%)	164 (21.3%)
Carbapenems	90 (1.7%)	49 (2.6%)	2 (0.9%)	13 (0.8%)	18 (2.8%)	8 (1.0%)
Monobactams	11 (0.2%)	1 (<0.1%)	0	2 (0.1%)	6 (0.9%)	2 (0.3%)
Aminoglycosides	208 (4%)	82 (4.4%)	9 (3.9%)	33 (1.9%)	56 (8.6%)	28 (3.6%)
Fluoroquinolones	1129 (21.6%)	244 (13.1%)	50 (21.5%)	651 (37.8%)	66 (10.2%)	118 (15.3%)
Penicillins	433 (8.3%)	138 (7.4%)	15 (6.4%)	164 (9.5%)	64 (9.9%)	52 (6.8%)
Penicillin plus β-lactamase inhibitors	1082 (20.7%)	456 (24.5%)	80 (34.3%)	179 (10.4%)	83 (12.8%)	284 (36.9%)
**Other**						
Glycopeptides	184 (3.5%)	74 (4.0%)	1 (0.4%)	17 (1%)	67 (10.3%)	25 (3.3%)
Metronidazole	301 (5.7%)	151 (8.1%)	2 (0.9%)	24 (1.4%)	96 (14.8%)	28 (3.6%)
Nitrofurans	60 (1.1%)	0	0	58 (3.4%)	0 (0)	2 (0.3%)
Sulphonamides/folic acid inhibitors	372 (7.1%)	9 (0.5%)	2 (0.9%)	346 (20.1%)	8 (1.2%)	7 (0.9%)
Macrolides	162 (3.1%)	88 (4.7%)	15 (6.4%)	9 (0.5%)	33 (5.1%)	17 (2.2%)

**Table 6:** Number of antibiotic agents prescribed for infection categories.

**Number of patients**	**Any infection 2708**	**Pneumonia 958**	**Tracheobronchitis 170**	**Urogenital infection 1132**	**Other infection 330**	**Uncertain infection 490**
**NUMBER OF ANTIBIOTIC AGENTS PRESCRIBED**, ***n*** **(%)**						
1	2184 (70.9%)	554 (57.8%)	136 (80.0%)	895 (79.1%)	221 (67.0%)	378 (77.0%)
2	669 (21.7%)	295 (30.8%)	30 (17.7%)	188 (16.6%)	72 (21.8%)	84 (17.1%)
3	165 (5.4%)	85 (8.9%)	2 (1.2%)	35 (3.1%)	21 (6.4%)	22 (4.5%)
4	49 (1.6%)	17 (1.8%)	2 (1.2%)	11 (1.0%)	12 (3.6%)	7 (1.4%)
5	11 (0.4%)	5 (0.5%)	0	2 (0.2%)	4 (1.2%)	0
6	3 (0.1%)	2 (0.2%)	0	1 (0.1)	0	0

**Figure 1:**
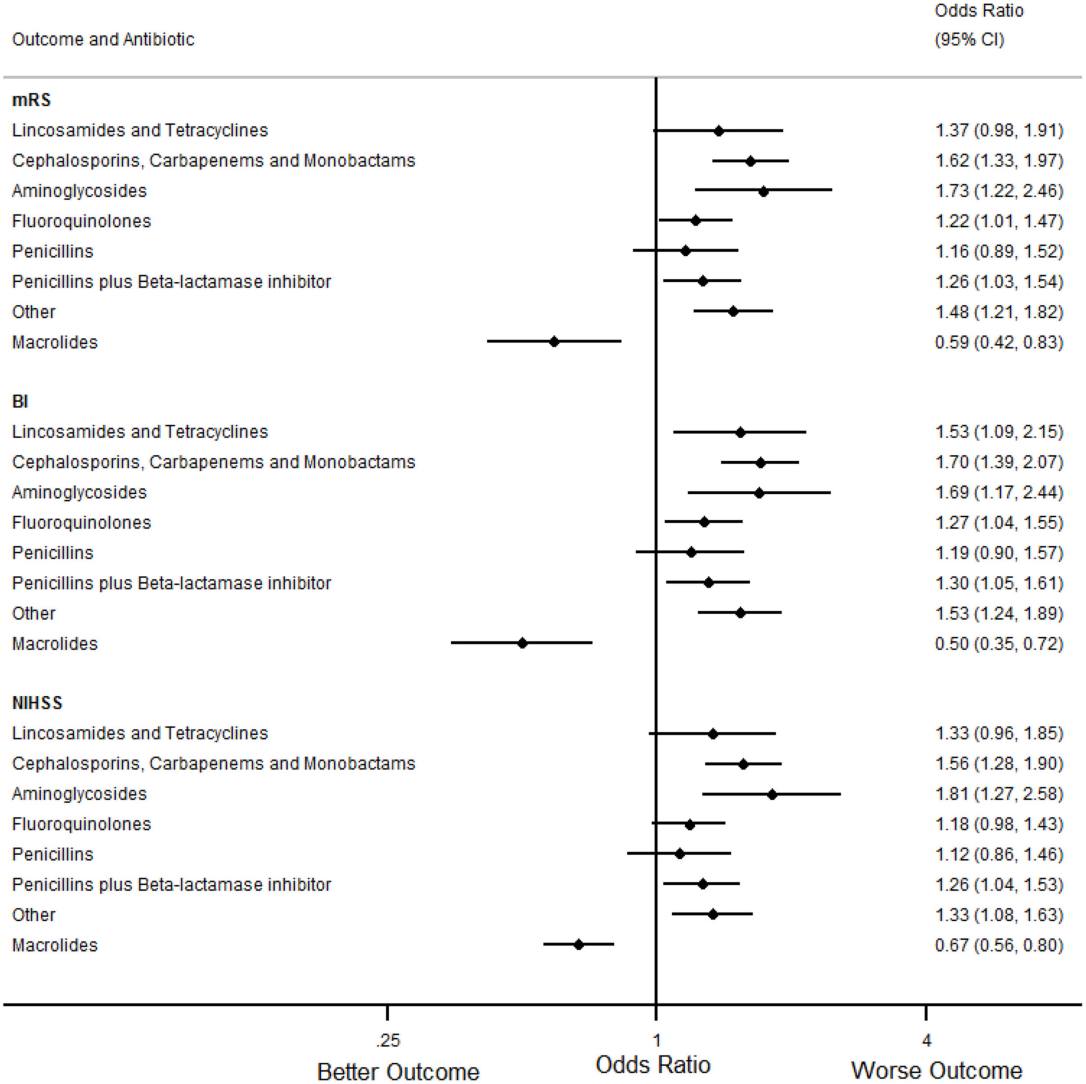
Forest plots illustrating the associations between antibiotic class and 90 day modified Rankin Scale (mRS), Barthel index (BI) and National Institutes of Health Stroke Scale (NIHSS) score for any infection, using multifactorial ordinal logistic regression. CI indicates Confidence Interval.

**Figure 2:**
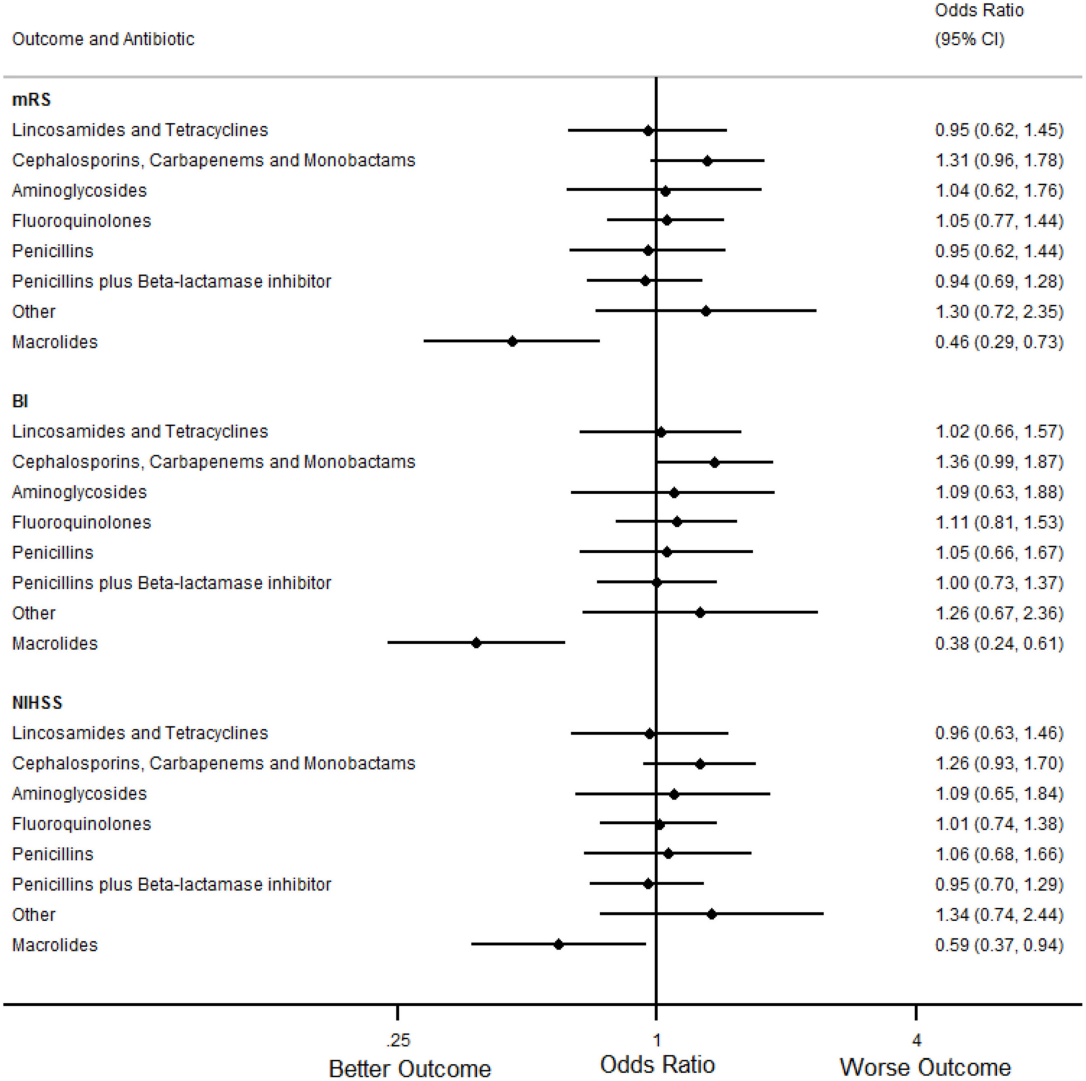
Forest plots illustrating the associations between antibiotic class and 90 day modified Rankin Scale (mRS), Barthel index (BI) and National Institutes of Health Stroke Scale (NIHSS) score for pneumonia, using multifactorial ordinal logistic regression. CI, Confidence Interval.

In the primary analyses (Table 7, Figures 1, 2), treatment with macrolides (5% of any infections; 9% of pneumonias) was independently associated with more favorable mRS distribution for any infection [OR (95% CI) = 0.59 (0.42 to 0.83), *p* = 0.004] and for pneumonia [OR (95% CI) = 0.46 (0.29 to 0.73), *p* = 0.001]. By contrast, unfavorable mRS distribution was independently associated with treatment of any infection with carbapenems, cephalosporins or monobactams [35% of any infections; OR (95% CI) = 1.62 (1.33 to 1.97), *p* < 0.001], penicillin plus β-lactamase inhibitor [35% of any infections; OR (95% CI) = 1.26 (1.03 to 1.54), *p* = 0.025] or with aminoglycosides [7% of any infections; OR (95% CI) = 1.73 (1.22 to 2.46), *p* = 0.002] (Figure 1).

**Table 7:** Multifactorial ordinal logistic regression for associations with unfavorable 90 day mRS.

	**Pneumonia (*****n*** **= 791 patients)**			**Any infection (*****n*** **= 2254 patients)**		
	***OR***	**95% CI**	***p***	***OR***	**95% CI**	***p***
**Antibiotic class**						
Lincosamides and tetracyclines	0.95	0.62 to 1.45	0.82	1.37	0.98 to 1.91	0.07
Cephalosporins, carbapenems, and monobactams	1.31	0.96 to 1.78	0.09	1.62	1.33 to 1.97	<0.001
Aminoglycosides	1.04	0.62 to 1.76	0.87	1.73	1.22 to 2.46	0.002
Fluoroquinolones	1.05	0.77 to 1.44	0.74	1.22	1.01 to 1.47	0.040
Penicillins	0.95	0.62 to 1.44	0.81	1.16	0.89 to 1.52	0.28
Penicillin plus β-lactamase inhibitor	0.94	0.69 to 1.28	0.68	1.26	1.03 to 1.54	0.025
Other^*^	1.30	0.72 to 2.35	0.38	1.48	1.21 to 1.82	<0.001
Macrolides	0.46	0.29 to 0.73	0.001	0.59	0.42 to 0.83	0.004
**Sex**						
Female	Ref			Ref		
Male	1.02	0.79 to 1.33	0.86	1.02	0.87 to 1.19	0.84
Increasing age (y)	1.05	1.04 to 1.06	<0.001	1.05	1.04 to 1.05	<0.001
Baseline NIHSS	1.11	1.08 to 1.15	<0.001	1.15	1.13 to 1.17	<0.001
**Infection subtype**						
Pneumonia				1.47	1.09 to 1.97	0.011
Tracheobronchitis				0.92	0.63 to 1.36	0.69
Urogenital				0.64	0.48 to 0.85	0.002
Other				0.77	0.57 to 1.02	0.097
Uncertain				1.17	0.86 to 1.58	0.31
**Treated with IV alteplase**						
No	Ref			Ref		
Yes	0.95	0.70 to 1.29	0.73	0.80	0.66 to 0.96	0.016
**Prior statin**						
No	Ref			Ref		
Yes	1.14	0.30 to 2.16	0.69	1.14	0.83 to 1.57	0.42
Unrecorded	1.71	0.96 to 3.02	0.07	1.62	1.18 to 2.23	0.003
**Smoking status**						
Non-smoker	Ref			Ref		
Smoker	1.29	0.67 to 2.16	0.69	1.21	0.83 to 1.57	0.42
Unrecorded	1.15	0.49 to 2.68	0.75	1.04	0.65 to 1.67	0.88
**Region**						
Australasia/ Africa	Ref			Ref		
Asia	0.85	0.39 to 1.85	0.68	1.14	0.68 to 1.92	0.62
Europe	0.85	0.41 to 1.77	0.66	1.10	0.67 to 1.79	0.72
America	0.92	0.44 to 1.96	0.84	1.03	0.62 to 1.69	0.91
Increasing year of study	0.97	0.90 to 1.04	0.41	1.00	0.96 to 1.04	0.88

* Other includes sulphonamides/folic acid inhibitors, metronidazole, glycopeptides and nitrofurans.

*mRS, modified Rankin Scale; OR, Odds ratio; CI, Confidence Interval; NIHSS, National Institutes of Health Stroke Scale; IV, intravenous*.

In secondary analyses, treatment with macrolides for any infection or pneumonia was also independently associated with more favorable BI and NIHSS (Tables 8, 9, Figures 1, 2). Treatment of any infection with carbapenems, cephalosporins or monobactams; penicillin plus β-lactamase inhibitor or with aminoglycosides was also independently associated with unfavorable BI and NIHSS (Figure 1).

**Table 8:** Multifactorial ordinal logistic regression for associations with worse 90 day BI.

	**Pneumonia (*****n*** **= 751 patients)**			**Any infection (*****n*** **= 2124 patients)**		
	***OR***	**95% CI**	***p***	***OR***	**95% CI**	***p***
**Antibiotic class**						
Lincosamides and tetracyclines	1.02	0.66 to 1.57	0.95	1.53	1.09 to 2.15	0.015
Cephalosporins, carbapenems and monobactams	1.36	0.99 to 1.87	0.05	1.70	1.39 to 2.07	<0.001
Aminoglycosides	1.09	0.63 to 1.88	0.75	1.69	1.17 to 2.44	0.005
Fluoroquinolones	1.11	0.81 to 1.53	0.54	1.27	1.04 to 1.55	0.02
Penicillins	1.05	0.66 to 1.67	0.84	1.19	0.90 to 1.57	0.23
Penicillin plus β-lactamase inhibitor	1.00	0.73 to 1.37	0.98	1.30	1.05 to 1.61	0.02
Other^*^	1.26	0.67 to 2.36	0.47	1.53	1.24 to 1.89	<0.001
Macrolides	0.38	0.24 to 0.61	<0.001	0.50	0.35 to 0.72	<0.001
**Sex**						
Female	Ref			Ref		
Male	1.02	0.78 to 1.33	0.91	1.03	0.88 to 1.21	0.73
Increasing age (y)	1.05	1.04 to 1.06	<0.001	1.05	1.04 to 1.06	<0.001
Baseline NIHSS	1.11	1.08 to 1.14	<0.001	1.14	1.12 to 1.16	<0.001
**Infection subtype**						
Pneumonia				1.52	1.12 to 2.07	0.007
Tracheobronchitis				0.79	0.53 to 1.17	0.24
Urogenital				0.65	0.49 to 0.88	0.005
Other				0.86	0.62 to 1.20	0.38
Uncertain				1.18	0.86 to 1.62	0.31
**Treated with IV alteplase**						
No	Ref					
Yes	0.85	0.62 to 1.16	0.31	0.70	0.58 to 0.84	<0.001
**Prior statin**						
No	Ref					
Yes	1.09	0.49 to 2.46	0.83	1.32	0.90 to 1.92	0.15
Unrecorded	1.38	0.78 to 2.47	0.27	1.17	0.84 to 1.61	0.35
**Smoking status**						
Non-smoker	Ref					
Smoker	1.06	0.56 to 2.01	0.85	0.81	0.55 to 1.19	0.29
Unrecorded	1.09	0.47 to 2.52	0.84	0.98	0.61 to 1.58	0.94
**Region**						
Australasia/Africa	Ref					
Asia	0.88	0.40 to 1.94	0.74	1.17	0.68 to 2.02	0.57
Europe	1.06	0.51 to 2.20	0.88	1.53	0.92 to 2.53	0.10
America	1.08	0.51 to 2.28	0.85	1.27	0.76 to 2.11	0.36
Increasing year of study	1.00	0.93 to 1.08	0.98	1.04	1.00 to 1.09	0.05

* Other includes sulphonamides/folic acid inhibitors, metronidazole, glycopeptides and nitrofurans.

*BI, Barthel Index; OR, Odds ratio; CI, Confidence Interval; NIHSS, National Institutes of Health Stroke Scale; IV, intravenous*.

**Table 9:** Multifactorial ordinal logistic regression for associations with worse 90 day NIHSS.

	**Pneumonia (*****n*** **= 781 patients)**			**Any infection (*****n*** **= 2215 patients)**		
	***OR***	**95% CI**	***P***	**OR**	**95% CI**	***p***
**Antibiotic class**						
Lincosamides and tetracyclines	0.96	0.63 to 1.46	0.86	1.33	0.96 to 1.85	0.09
Cephalosporins, carbapenems, and monobactams	1.26	0.93 to 1.70	0.14	1.56	1.28 to 1.90	<0.001
Aminoglycosides	1.09	0.65 to 1.85	0.74	1.81	1.27 to 2.58	0.001
Fluoroquinolones	1.01	0.74 to 1.38	0.95	1.18	0.98 to 1.43	0.09
Penicillins	1.06	0.68 to 1.66	0.79	1.12	0.86 to 1.46	0.40
Penicillin plus β-lactamase inhibitor	0.95	0.70 to 1.29	0.73	1.26	1.04 to 1.53	0.02
Other^*^	1.34	0.74 to 2.44	0.34	1.33	1.08 to 1.63	0.006
Macrolides	0.59	0.37 to 0.94	0.03	0.67	0.56 to 0.80	0.007
**Sex**						
Female	Ref			Ref		
Male	1.12	0.86 to 1.45	0.41	1.08	0.92 to 1.26	0.34
Increasing age (y)	1.04	1.02 to 1.05	<0.001	1.03	1.02 to 1.04	<0.001
Baseline NIHSS	1.14	1.11 to 1.18	<0.001	1.18	1.16 to 1.20	<0.001
**Infection subtype**						
Pneumonia				1.57	1.17 to 2.11	0.003
Tracheobronchitis				0.95	0.65 to 1.38	0.78
Urogenital				0.68	0.51 to 0.91	0.008
Other				0.84	0.61 to 1.14	0.27
Uncertain				1.15	0.85 to 1.56	0.37
**Treated with IV alteplase**						
No	Ref					
Yes	0.93	0.69 to 1.27	0.66	0.78	0.65 to 0.93	0.007
**Prior statin**						
No	Ref					
Yes	1.06	0.56 to 2.00	0.87	1.00	0.73 to 1.38	0.99
Unrecorded	1.51	0.86 to 2.64	0.15	1.24	0.91 to 1.69	0.18
**Smoking status**						
Non-smoker	Ref					
Smoker	1.17	0.63 to 2.20	0.62	1.08	0.74 to 1.56	0.70
Unrecorded	1.04	0.45 to 2.39	0.93	0.97	0.60 to 1.54	0.88
**Region**						
Australasia/Africa	Ref					
Asia	1.19	0.55 to 2.59	0.66	1.59	0.95 to 2.64	0.08
Europe	0.97	0.47 to 2.01	0.94	1.18	0.73 to 1.90	0.51
America	1.05	0.50 to 2.22	0.90	1.16	0.71 to 1.90	0.54
Increasing year of study	0.96	0.90 to 1.04	0.32	0.99	0.95 to 1.03	0.57

* Other includes sulphonamides/folic acid inhibitors, metronidazole, glycopeptides and nitrofurans.

*OR, Odds ratio; CI, Confidence Interval; NIHSS, National Institutes of Health Stroke Scale; IV, intravenous*.

A sensitivity analysis of the primary and secondary analyses included interaction terms between each antibiotic class and baseline stroke severity using the NIHSS score. This was to examine whether any apparent association between antibiotic class and change in outcome could be due to an interaction with stroke severity. We did not find evidence of any interaction between any of the antibiotic classes and baseline stroke severity, including for macrolides (data not shown).

Forty-five percent of infections treated with macrolides were with macrolides alone. In the remainder, macrolides were most frequently combined with cephalosporins, fluoroquinolones or penicillin plus β-lactamase inhibitor. By contrast, 70% of infections treated with penicillin plus β-lactamase inhibitor were with monotherapy, the remainder were combined most often with fluoroquinolones or cephalopsorins. Fifty-nine percent of infections treated with cephalosporins were with monotherapy, the remainder used combination treatment most often with metronidazole, fluoroquinolones or penicillin plus β-lactamase inhibitor.

## Discussion

We found that treatment with macrolides was independently associated with a favorable clinical outcome in any infection or pneumonia complicating stroke. Macrolides were only used in a minority of post-stroke infections and were mainly used as combination therapy with other antibiotics. By contrast, treatment with cephalosporins, carbapenems or monobactams; or penicillin plus β-lactamase inhibitors, was consistently associated with unfavorable outcome in any infection, but not pneumonia. These two classes were each used in the treatment of over one third of post-stroke infections, mainly as monotherapy. Aminoglycosides were also associated with unfavorable outcome when used to treat any infection but accounted for a small proportion of the antibiotic classes used. These associations were consistently observed with all three clinical outcome measures in the primary and secondary analyses.

Genito-urinary infections were marginally more frequent than pneumonia in our study. This is in keeping with a recent study using both clinician-diagnosed and expert panel-adjudicated diagnosis of urinary tract infection and pneumonia (3) and estimates from meta-analysis of stroke studies (2, 24, 25). However, pneumonia is associated with substantial risk of death and disability compared to urinary tract infections (2) and is regarded as the major research and clinical priority amongst stroke-associated infections. Our data accord with this as only pneumonia, but not other infection sub-types, was independently associated with worse clinical outcomes.

Our retrospective study has several limitations which necessitate caution when interpreting the findings. First, choice of antibiotic class will have been influenced by study- and individual-level factors, leading to effect modification and confounding by indication. We were unable to include a trial-identifier in our analyses to account for non-specific differences in study populations. Perceived severity of infection will likely have been a determinant of choice of antibiotic class, along with availability of oral, enteral or parenteral routes, local antibiotic policy and allergy status. Bacterial species implicated in aspiration pneumonia have changed in recent years as anaerobes are less frequently isolated (26). Changing patterns of antimicrobial susceptibility may also have influenced regional and local prescribing. Plasma C-reactive protein was not sufficiently available for the regression analyses, but may still have been used by treating clinicians to inform antibiotic choice. Second, details of route, dose and duration of treatment were not available and may also have influenced the findings. Third, the criteria used for diagnosis of infection were not available and will have varied between different units, leading to under- or over-diagnosis and therefore selection bias. There were also very limited microbiological data available, reflected by the need for an uncertain infection category, where infection was suspected (and antibiotics initiated) yet a definite site was not recorded. As there are no ubiquitous, validated criteria for diagnosing common post-stroke infections such as pneumonia, and positive microbiological data are infrequent (27), this reflects real-life practice. Finally, our categorization of antibiotic class required a balance between prevalence and mechanisms of action, to avoid over-fitting the regression models. We were therefore unable to further sub-divide antibiotic classes, such as creating a separate category for carbapenems and for monobactams, or by generation of drug (e.g., cephalopsorins).

In randomized trials of prophylactic antibiotics in acute stroke, several classes of antibiotics commonly used for post-stroke infections, including cephalosporins, penicillin plus β-lactamase inhibitors and fluoroquinolones, did not appear to prevent pneumonia complicating stroke, even in patients selected at higher-risk (3, 5, 15–17). This raises the possibility that some antibiotics are not optimally effective against pneumonia complicating stroke (18). Whilst this may have contributed to our observations, the limitations of our study necessitate that such hypothesis-generation requires testing in appropriately designed studies.

Antibiotics used to treat post-stroke infections could influence clinical outcome by three main mechanisms. First, antimicrobial spectrum could explain differential outcomes between antibiotic classes. Stroke-associated pneumonia is predominantly associated with aerobic Gram-negative bacilli (e.g., *Klebsiella pneumoniae* and *Escherichia coli*) and Gram-positive cocci (e.g., *Stapylococcus spp*.) (27). Whilst the antimicrobial spectrum of β-lactam antibiotics varies, penicillin plus β-lactamase inhibitors and third generation cephalosporins are broadly comparable despite differences in coverage of *Pseudomonas spp*. By contrast, macrolides are mainly active against Gram-positive organisms (e.g., *Streptococcus pneumoniae*), with limited activity against Gram-negative organisms. It therefore seems unlikely that any beneficial effect of macrolide therapy for stroke-associated infections (including stroke-associated pneumonia) would be explained on the basis of anti-microbial spectrum. However, macrolides improve coverage of atypical organisms in community acquired pneumonia (CAP), and have synergistic effects combined with β lactam agents, which could be relevant in stroke-associated pneumonia (28). Indeed, macrolides reduced in-patient mortality rates in hospitalized, severe CAP when added to β-lactam antibiotics (29).

Second, several antibiotic classes have immuno-modulatory effects which could either facilitate host immune responses to bacterial challenge, or limit excessive inflammation in the lung. Macrolides have a range of immuno-modulatory effects (30), suppressing innate responses and inducing Th1 to Th2 shift. In a mouse model of pneumococcal pneumonia, addition of azithromycin to ceftriaxone induced specific changes in immune-checkpoint ligands (e.g., up-regulation of CD86 and MHC II in neutrophils and CD11b^+^CD11c^+^ macrophages) and receptors (e.g., down-regulation of CTLA-4) in bronchoalveolar lavage fluid, which may have contributed to the observed increased survival (31).

Third, peripherally administered antibiotics could have protective or deleterious effects on evolving cerebral infarction, reparative processes, or both, particularly as they are frequently prescribed within the first week after stroke onset. In experimental stroke, in the absence of infection, macrolides administered peripherally after middle cerebral artery occlusion are consistently protective; reducing infarct volume, blood brain barrier damage, markers of oxidative stress, infiltration of circulating immune cells and early neurological deficit (12, 14, 32–34). By contrast, peripheral administration of third generation cephalosporins in experimental stroke has revealed conflicting findings in different studies (10, 35, 36). However, in humans, cerebrospinal-fluid penetration of macrolides and β-lactam antibiotics is generally poor, and highest for fluoroquinolones and metronidazole (37).

## Conclusion

In this retrospective analysis, treatment of any infection or pneumonia within 2 weeks of stroke with macrolides was independently associated with a favorable clinical outcome. By contrast, treatment of any infection with cephalosporins, carbapenems or monobactams; or penicillin plus β-lactamase, was independently associated with unfavorable outcome. Although there is a risk of residual confounding and bias, these data warrant further study in an appropriately designed prospective study or controlled trial evaluation.

## Data Availability

The datasets used in the analyses for this manuscript were obtained from the Virtual International Stroke Trials Archive (VISTA)-Acute (www.virtualtrialsarchives.org/vista). Requests to access the dataset from qualified researchers trained in human subject confidentiality protocols may be sent to VISTA-Acute at vista.acute@glasgow.ac.uk.

## Ethics Statement

All procedures performed in studies involving human participants were in accordance with the ethical standards of the institutional and/or national research committee and with the 1964 Helsinki declaration and its later amendments or comparable ethical standards. The study protocol was approved by the VISTA-Acute Steering Committee, as part of the Data Request Form submission process. Informed consent was not required as anonymized historical patient data from randomized trials were being used.

## Informed Consent

Informed consent was not required as anonymized historical patient data from randomized trials were being used.

## Author Contributions

CS conceived the study and was involved in study design, data acquisition, analysis, interpretation, drafting the manuscript, final approval for submission. CH and AV were involved in study design, data acquisition, analysis, interpretation, drafting the manuscript, final approval for submission. AJ was involved in study design, data acquisition, analysis, interpretation, drafting the manuscript, final approval for submission. WW, PN, DvdB, LK, JM, MW, and AM were involved in study design, interpretation, drafting the manuscript, final approval for submission. All authors agree to be accountable for all aspects of the manuscript.

### Conflict of Interest Statement

The authors declare that the research was conducted in the absence of any commercial or financial relationships that could be construed as a potential conflict of interest.
